# Intelligent Extraction of Salient Feature From Electroencephalogram Using Redundant Discrete Wavelet Transform

**DOI:** 10.3389/fnins.2022.921642

**Published:** 2022-06-01

**Authors:** Xian-Yu Wang, Cong Li, Rui Zhang, Liang Wang, Jin-Lin Tan, Hai Wang

**Affiliations:** ^1^State Key Laboratory of Integrated Service Networks, Xidian University, Xi’an, China; ^2^Academy of Space Electronic Information Technology, Xi’an, China; ^3^Shaanxi Academy of Aerospace Technology Application Co., Ltd., Xi’an, China; ^4^School of Aerospace Science and Technology, Xidian University, Xi’an, China

**Keywords:** EEG feature analysis, brain-computer interface, redundant representation, translation invariance, wavelet transform, deep learning, artificial intelligence

## Abstract

At present, electroencephalogram (EEG) signals play an irreplaceable role in the diagnosis and treatment of human diseases and medical research. EEG signals need to be processed in order to reduce the adverse effects of irrelevant physiological process interference and measurement noise. Wavelet transform (WT) can provide a time-frequency representation of a dynamic process, and it has been widely utilized in salient feature analysis of EEG. In this paper, we investigate the problem of translation variability (TV) in discrete wavelet transform (DWT), which causes degradation of time-frequency localization. It will be verified through numerical simulations that TV is caused by downsampling operations in decomposition process of DWT. The presence of TV may cause severe distortions of features in wavelet subspaces. However, this phenomenon has not attracted much attention in the scientific community. Redundant discrete wavelet transform (RDWT) is derived by eliminating the downsampling operation. RDWT enjoys the attractive merit of translation invariance. RDWT shares the same time-frequency pattern with that of DWT. The discrete delta impulse function is used to test the time-frequency response of DWT and RDWT in wavelet subspaces. The results show that DWT is very sensitive to the translation of delta impulse function, while RDWT keeps the decomposition results unchanged. This conclusion has also been verified again in decomposition of actual EEG signals. In conclusion, to avoid possible distortions of features caused by translation sensitivity in DWT, we recommend the use of RDWT with more stable performance in BCI research and clinical applications.

## Introduction

As an important tool for physiological disease diagnosis and brain-computer interface (BCI) research (Khan et al., 2014), the research on EEG signal processing is a hot issue in academic and engineering field (Rahman et al., 2021). EEG can be used to diagnosis diseases such as encephalopathy (Jacob et al., 2021), epilepticus (Miki et al., 2019), and depression (Sharma et al., 2018). EEG is a kind of electrical signal obtained from human scalp, which has the advantages of convenient acquisition and low cost. Because it is a non-invasive detection method, although the safety is high, the information obtained is relatively weak in energy. In addition, the actual measurements often contain interference from other physiological processes (e.g., EMG) and a variety of measurement noise (e.g., base-line wander). In order to extract the characteristic components that can truly represent the brain activity, it is essential to use signal processing methods to process EEG signals (Wang et al., 2019).

In EEG signal processing, fast Fourier transform (FFT) is the most classical analysis method besides statistical analysis (Chen et al., 2016; Li et al., 2021; Li and Chen, 2021). Because FFT uses a trigonometric basis composed of triangular waves, it is extremely suitable for analyzing periodic components (Chen et al., 2021). However, it cannot effectively analyze and extract the non-stationary components in EEG. In view of the shortcomings of FFT, some improved methods with good performance have appeared in recent years (Guarascio and Puthusserypady, 2017; Rajeswari and Jagannath, 2017; Huang et al., 2019), such as short time Fourier transform (STFT) (Demetgul et al., 2022), time-frequency representation (Sheela and Puthankattil, 2021), and sparse representation (Chen et al., 2021). Among the massive novel signal decomposition methods reported in the literature, wavelet decomposition is the most effective one because of its strict mathematical foundation and high computational efficiency (Huang et al., 2021). Both of continuous wavelet transform (CWT) and discrete wavelet transform (DWT) are widely been applied to EEG signal processing in clinical diagnosis, and DWT receives more applications because it is more convenient to be used (Real et al., 2014; Cordes et al., 2021).

In state-of-the-art literature, the understanding of EEG not only includes the step of signal decomposition, but also incorporates the utilization of decision methods to identify or classify the analysis results (Cao et al., 2019; Gu et al., 2021). Currently, such decision methods are mainly based on statistical learning methods, and artificial intelligence is the main development direction (Amin et al., 2019). Huang et al. (2020) proposed an intelligent method, based on the combination of convolutional neural network and sparse representation, to enable automatic EEG classification. Khademi et al. (2022) proposed a hybrid AI-based model for motor imagery EEG signal classification based on CNN and LSTM. Shoji et al. (2021) studied the detecting of abnormal patterns and electrodes in EEGs by using a model inspired by CNN.

Although wavelet transform has achieved great successes in EEG signal processing, its shortcomings have been revealed constantly (Luo et al., 2020). One of the most serious problems is translational variability of DWT. This problem originates from the numerical implementation of DWT. To decompose a signal, the time-frequency atoms of DWT originate from a prototype scaling function and prototype wavelet function. A tree-structured filter bank is employed to implement the multiscale decomposition. Within the filterbank, downsampling, and upsampling operators are utilized, which results in the number of representation coefficients in the wavelet subspace being less than the number of reconstructed signal coefficients. Therefore, the analysis results of the conventional DWT are sensitive to the translation of the input signal. This phenomenon is known in the literature as translation variability (Li et al., 2021). This phenomenon exists not only in DWT, but also in wavelet packet decomposition (WPT), because the latter also uses downsampling operations in the decomposition process. This effect is hazardous for salient feature extraction of EEG measurements. To solve this problem, a redundant discrete wavelet transform (RDWT) that removes the downsampling and upsampling operations can be used. In this paper, we take wavelet transform as an example to study the effect of TV on EEG feature extraction. The sensitivity of DWT and RDWT to signal shift is analyzed. It is pointed out that DWT is actually a linear variant system, and there are extra noises in its wavelet subspaces. The above conclusions are verified by numerical simulation and analysis of real EEG signals.

## Materials and Methods

### Fundamentals of Discrete Wavelet Transform and Redundant Discrete Wavelet Transform

**FIGURE 1:**
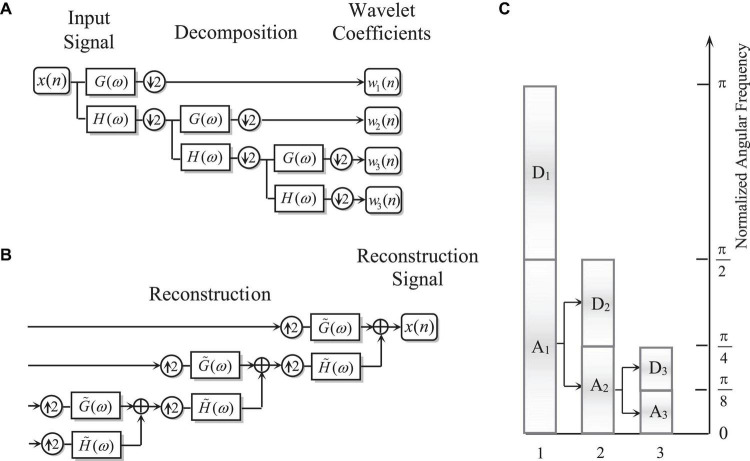
**(A)** The decomposition process of wavelet transform; **(B)** the reconstruction process of wavelet transform; and **(C)** the theoretical spectral passing band of wavelet transform.

During the past decades, the concept of wavelet emerges as a prototype function ψ(*t*)with fast decaying behavior. The wavelet transform is conducted between the inner product of an input signal and wavelet atoms. The latter is formed by scaling and shifting the prototype function and it is denoted as ψ(*t*).


(1) $$c_{j}\,\left(k\right)=2^{j/2}\,\int_{-\infty }^{+\infty }x\,\left(t\right)\,\psi \,\left(2^{j}\,\text{t-k}\right)\,dt,$$

where *c*_*j*_(*k*) denotes the *k*-th coefficient at *J*-level decomposition stage. In the discrete wavelet transform (DWT), the analog signal *x*(*t*) needs to be replaced by a discretized series *x*(*n*) of length *N*. In a *J*-level DWT decomposition, a scaling subspace (*w*_*j* + 1_(*n*)) and *J* wavelet subspaces ({*w*_*i*_(*n*)|1≤*i*≤*J*}) are generated. The theoretical spectral passing band (SFB) of *w*_*i*_(*n*) is denoted by*TSPB*{*w*_*j*_} = [*f*_*s*_/2^*j* + 1^,*f*_*s*_/2^*j*^]. The number of samples at *w*_*i*_(*n*) is *N*/2^*i*^, and the number of samples at *w*_*j* + 1_(*n*) is *N*/2^*J*^. Due to the two scale relationship of the wavelet function, the numerical implementation of wavelet can be achieved by an iterative process (Figures 1A,B). In order to make the total number of samples of the wavelet sequence generated by DWT the same as the number of samples of the input signal, down-sampling of the decomposition process and up-sampling of the reconstruction process are widely used, which is also referred as a typical example the multi-rate signal processing (Figure 1C). Although this can improve the efficiency of the algorithm, it also causes some adverse effects.

### Shortcoming of Discrete Wavelet Transform

With the increase of the number of wavelet decomposition levels, the number of coefficients of the subspace decreases, while the total length of the reconstructed subspace remains unchanged. This means that longer data needs to be recovered with fewer coefficients, which results in a significantly greater chance of errors in the reconstructed signal. One of the shortcomings is translation variability, which exists in every wavelet subspace of DWT. Wang et al. (2010) and Sahoo et al. (2021) used multi-harmonic signals to verify the influence of the TV property on DWT analysis results. In order to suppress the side effect of translation variability on the subspace reconstruction results, the main measure is to increase the number of wavelet coefficients of the decomposition process, which means that the overcomplete expansion is introduced. Redundant discrete wavelet transform (RDWT) shares the same wavelet bases with DWT. The only difference is the removal of the upsampling and downsampling operations in the filterbank.

## Results

### Demonstrations of Discrete Wavelet Transform and Redundant Discrete Wavelet Transform in Delta Impulse Decomposition

To test the translation sensitivity of DWT and RDWT, we employ discrete delta impulse function δ(*n*−*L*)as input of wavelet decomposition. The mathematical definition of δ(*n*−*L*) is defined as in Equation (2). The output can be regarded as wavelet functions in each wavelet subspace.


(2) $$\delta \,\left(n-L\right)=\left\{ \begin{array}{c} 1,f\,o\,r\,n=L\\ 0,f\,o\,r\,n\neq L \end{array}\right.$$

#### Scheme of Signal Decomposition

**FIGURE 2:**
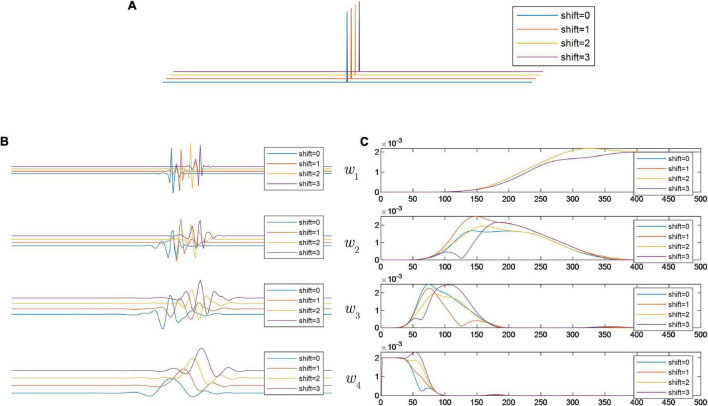
**(A)** Shifted versions of discrete delta impulse function; **(B)** time domain waveforms of wavelet subspaces generated by DWT; and **(C)** the FFT spectra of the decomposed wavelet subspaces generated by DWT.

In this sub-section, a numerical simulation is provided to illustrate the TV of DWT. A discrete delta impulse function is synthesized as input of DWT. In the simulated signal, the parameters are set as *N* = 1000 and *L* = 500. The time domain waveform of δ(*n*−*L*) is plotted in Figure 2A. A J-level wavelet decomposition is used to decompose δ(*n*−*L*).


(3) $$\delta \,\left(n-L\right)\,\left\{ \begin{array}{c} \overset{D\,W\,T}{⟶}w_{J,0}\,\left(n\right),w_{J,1}\,\left(n\right),\ldots ,w_{J,L}\,\left(n\right)\\ \overset{R\,D\,W\,T}{⟶}w_{J,0}^{r}\,\left(n\right),w_{J,1}^{r}\,\left(n\right),\ldots ,w_{J,3}^{r}\,\left(n\right) \end{array}\right.$$

To show the differences in the decomposition results, we compare the subspaces in the frequency domain. The Daubechies-8 (DB8) basis function, which has been widely used in engineering applications, is selected for DWT and the decomposition stage is set as 3. Without loss of generality, the sampling frequency in the numerical simulation is set to 1,000 samples per second. Decomposition results of the wavelet subspaces are shown in Figure 2B. From the decomposition results, it can be seen that when the input signal is shifted, the results of wavelet transform are quite different. Correspondingly, we plot the fast Fourier spectrum of each decomposition result (Figure 2C). From the FFT spectra, it can be found that differences among these subspaces are not negligible. It is generally believed that each subspace of the wavelet transform has a well-defined spectral passing band, but from the numerical simulation, due to the existence of translational variability, this property is conditional. On the other hand, it can also be found that this translation variability has some periodicity. For example, as shown in the FFT spectra of *w*_1_(*n*), four sequentially shifted inputs correspond to two different spectrograms. The periodicity of TV for *w*_*j*_ (1≤*j*≤*J* + 1) can be summarized as below.


(4) P⁢e⁢r⁢i⁢o⁢d⁢i⁢c⁢i⁢t⁢y⁢o⁢f⁢T⁢V⁢f⁢o⁢r⁢wj={2j⁢   1≤j≤J2j⁢j=J+1

In order to investigate the effect of translations of input signal on the decomposition results, another integer variable $\tilde{L}$ is introduced. That is, the input signal is set as shifted versions of δ(*n*−*L*) to the right by a specific sample point $\tilde{L}$. By applying the DWT and RDWT, we have the following decomposition results.


(5) $$\delta \,\left(n-L-\tilde{L}\right)\,\left\{ \begin{array}{c} \overset{D\,W\,T}{⟶}{\tilde{w}}_{0,\tilde{L}}\,\left(n\right),{\tilde{w}}_{1,\tilde{L}}\,\left(n\right),{\tilde{w}}_{2,\tilde{L}}\,\left(n\right),{\tilde{w}}_{3,\tilde{L}}\,\left(n\right)\\ \overset{R\,D\,W\,T}{⟶}{\tilde{w}}_{0,\tilde{L}}^{r}\,\left(n\right),{\tilde{w}}_{1,\tilde{L}}^{r}\,\left(n\right),{\tilde{w}}_{2}^{r}\,\left(n\right),{\tilde{w}}_{3,\tilde{L}}^{r}\,\left(n\right) \end{array}\right.$$

**FIGURE 3:**
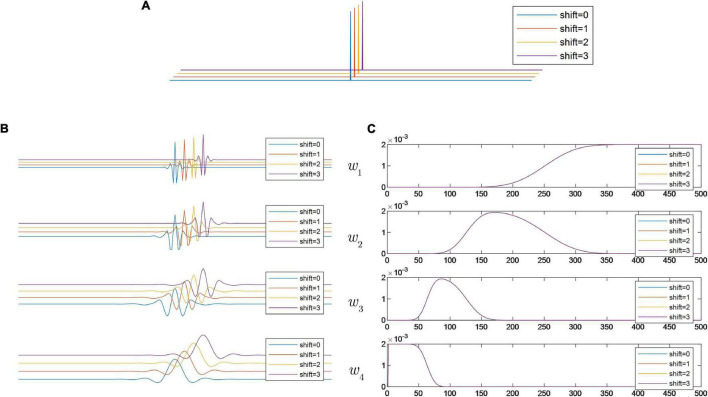
**(A)** Shifted versions of discrete delta impulse function; **(B)** time domain waveforms of wavelet subspaces generated by RDWT; and **(C)** FFT spectra of the decomposed wavelet subspaces generated by RDWT.

To investigate the translation sensitivity of the two wavelet transforms, we should compare the similarity between decomposition results in Equation (3) and those in Equation (5). As shown in Figure 3, when the input signal is shifted in turn, the shape of both the time domain waveform and the FFT spectrum of the wavelet subspace are exactly congruent. It can be inferred that in this case, the wavelet transform has a well-defined definition of the spectral passing band. By comparing the analysis results of Figures 2C, 3C, it is clear that all the decomposition results of DWT can be regarded as distorted, because no wavelet subspace has the same spectrum as the results of Figure 3C.

### Demonstrations of Redundant Discrete Wavelet and Redundant Discrete Wavelet Transform in Electroencephalogram Signal Decomposition

#### Description of Dataset

In order to evaluate the effect of translational variability on the decomposition of actual EEG signals, test data from clinical patients are used. The data for the analysis were obtained from the EEG dataset delivered publicly on the Internet by Neurology and Sleep Centre, Hauz Khas, New Delhi. These recorded signals were sampled at a frequency of 200 Hz.

#### Comparisons of Redundant Discrete Wavelet and Redundant Discrete Wavelet Transform in Electroencephalogram Signal Analysis

**FIGURE 4:**
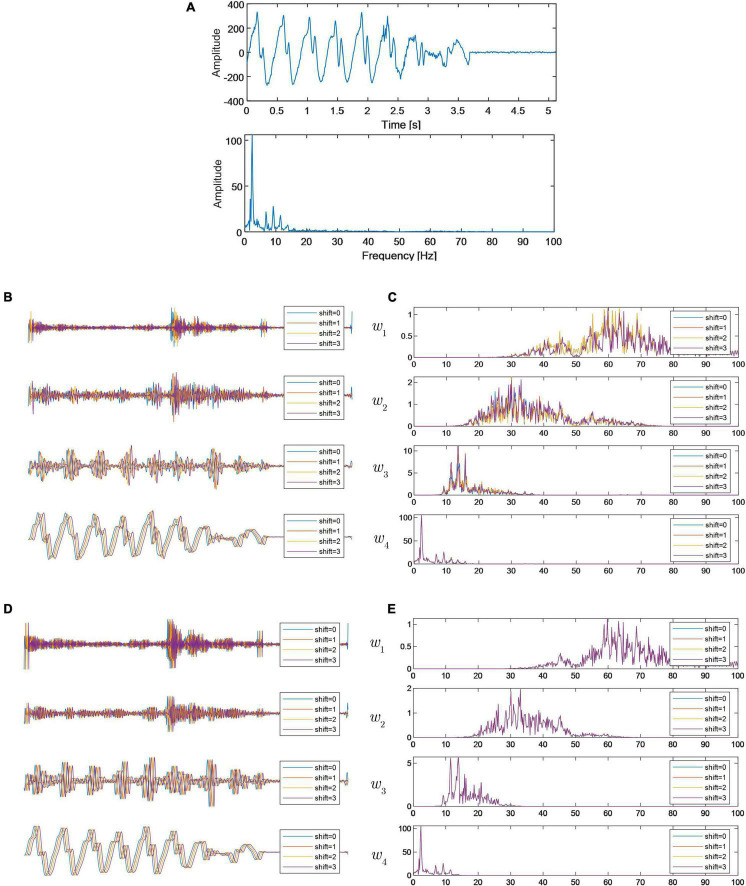
**(A)** Time domain waveform and FFT spectrum of the investigated EEG signal; **(B)** wavelet subspaces generated by DWT; **(C)** FFT spectra of wavelet subspaces generated by DWT; **(D)** wavelet subspaces generated by RDWT; and **(E)** FFT spectra of wavelet subspaces generated by RDWT.

The EEG signal investigated in this subsection was collected from ictal stages. The time domain series and its FFT spectrum are shown in Figure 4A. Since the anti-aliasing filter is used in the signal acquisition process, the signal characteristics are mainly concentrated in the lower frequency band.

For comparison, the input signal is sequentially shifted by one sample for a total of four times, and the translated signals are decomposed by DWT and RDWT. Wavelet subspaces and their FFT spectra, derived by DWT, are shown in Figures 4B,C. In each wavelet subspace, time domain waveforms, derived from translations of the signal, are different in shape. The energy in the high frequency band *w*_1_ is comparatively small, but their differences are prominent. Although time domain waveforms in the low frequency band *w*_4_ are also affected by TV, the differences are relatively small. Wavelet subspaces and their FFT spectra, derived by RDWT, are shown in Figures 4D,E. The decomposition results show that the waveforms of the decomposition results of each shifted input signal are exactly congruent due to the exact shift invariance of RDWT. Summarizing the above analysis results, we can see that TV will cause additional noise in the wavelet subspace, and the actual SPB is different from the theoretical SPB. The influence of TV on the transient feature extraction in the high frequency subspace is particularly significant. Therefore, in the actual use of wavelet transform, we should try our best to avoid the adverse effects of TV on the analysis results. In other words, we recommend using RDWT. In the literature, RDWT has been applied successfully in biomedical signal processing (Zeng et al., 2020; Kumar et al., 2021; Madan et al., 2022). However, it should be pointed out that the TV property of DWT here is for the wavelet subspace, and the wavelet transform satisfies the perfect reconstruction property for the whole signal.

## Discussion

### Comparison on Essential Differences of Redundant Discrete Wavelet and Redundant Discrete Wavelet Transform

Traditionally, scholars believe that the nature of the wavelet transform is completely determined by the wavelet basis function. In this paper, we show that the properties of the wavelet transform are also affected by the structure of the filter bank. This issue has rarely been studied systematically.

From the comparison of the above analysis results, it is consolidated that TV has a significant impact on the analysis results of discrete wavelet transform. DWT can be regarded is an example of linear time variant system, while RDWT is an example of LTI system. Therefore, even if they have the same time-frequency pattern for the same input signal, the decomposition results in the same subspace are still different.

Although DWT and RDWT are both linear transforms, their sensitivities to input signal shifts are quite different. This property is referred to the linear time-invariant (LTI) property in the theory of signals and systems. Let *x*(*t*) and *y*(*t*) be the input and output of a linear transform.


(6) $$x\,\left(t\right)\overset{L\,i\,n\,e\,a\,r\,S\,y\,s\,t\,e\,m}{⟶}y\,\left(t\right)$$

Let τ be the translation in the time domain, the response of *x*(*t*−τ) through the linear system is denoted by $\tilde{y}\,\left(t\right)$.


(7) $$x\,\left(t-\tau \right)\overset{L\,i\,n\,e\,a\,r\,S\,y\,s\,t\,e\,m}{⟶}\tilde{y}\,\left(t\right)$$

If the response $\tilde{y}\,\left(t\right)$ is a delayed version of *y*(*t*) in time, i.e., $\tilde{y}\,\left(t\right)=y\,\left(t-\tau \right)$ for arbitrary time delay τ, the system is known as linear time invariant (LTI). While for a linear time variant (LTV) system, the above equation cannot be satisfied. According to this definition and the results of numerical simulation, we can conclude that DWT is a concrete example of LTV and RDWT is a concrete example of LTI. Therefore, DWT can be regarded as an approximation of RDWT, which has higher computational efficiency, but it causes distortion on the decomposition results.

In this paper, we take the DB8 wavelet basis as an example to show that DWT has the problem of TV. The DB8 wavelet basis is chosen because it is widely used in biomedical signal processing. However, without loss of generality, for any basis function, as long as the DWT decomposition method is used, TV is inevitable, even if the support length of the wavelet basis is increased.

### Alternative Solutions to Enhance Time Invariance

Although RDWT satisfies the exact translation invariant property in signal decomposition, it also greatly increases the amount of computation. Based on the beneficial idea of improving TV by redundancy, many approximate translation invariant wavelet transforms have been developed. The most typical example is the dual-tree complex wavelet transform, which uses one times redundancy to achieve almost perfect translation invariance (Celik and Tjahjadi, 2010; Huang et al., 2021). On the other hand, because it is difficult to construct a new wavelet basis in the time domain, scholars are more and more inclined to design wavelet transform directly in the frequency domain (George et al., 2020). Typical examples include the harmonic wavelet transform (Musha and Kumazawa, 2007) and the rational dilation wavelet transform (Sharma et al., 2021). No matter what kind of construction idea, it has been proved to have a good performance in the practice of EEG signal processing.

### Translation Variability in Convolutional Neural Network

The pooling operation in CNN can be regarded as the down-sampling operation in wavelet transform. If the pooling operation is deleted in the CNN, the CNN also has the property of translation invariance, but this increases the complexity of CNN training. On the other hand, a large number of practices have shown that for images, the pooling operation does not cause a significant decline in classification accuracy.

## Data Availability Statement

Publicly available datasets were analyzed in this study. This data can be found here: https://www.researchgate.net/publication/308719109_EEG_Epilepsy_Datasets.

## Author Contributions

HW conceived and designed the method, reviewed, and edited the manuscript. X-YW and CL performed the experiment. X-YW, RZ, LW, and J-LT wrote the manuscript. All authors read and approved the manuscript.

## Conflict of Interest

RZ, LW, and J-LT were employed by Shaanxi Aerospace Technology Application Research Institute Co., Ltd. The remaining authors declare that the research was conducted in the absence of any commercial or financial relationships that could be construed as a potential conflict of interest.

## Publisher’s Note

All claims expressed in this article are solely those of the authors and do not necessarily represent those of their affiliated organizations, or those of the publisher, the editors and the reviewers. Any product that may be evaluated in this article, or claim that may be made by its manufacturer, is not guaranteed or endorsed by the publisher.
